# Case Report: Beyond chronicity: sustained multidimensional response to spinal cord stimulation three decades after traumatic spinal cord injury

**DOI:** 10.3389/fpain.2026.1969355

**Published:** 2026-10-07

**Authors:** Andrea Blažević, Petar Marčinković, Fadi Almahariq, Sven Krušić, Domagoj Dlaka, Darko Chudy, Marina Raguž, Tonko Marinović

**Affiliations:** 1 Department of Neurosurgery, Dubrava University Hospital, Zagreb, Croatia; 2 Faculty of Education and Rehabilitation Sciences, University of Zagreb, Zagreb, Croatia; 3 School of Medicine, Catholic University of Croatia, Zagreb, Croatia; 4 School of Medicine, University of Zagreb, Zagreb, Croatia; 5 Medicine of Sports and Exercise Chair, Faculty of Kinesiology, University of Zagreb, Zagreb, Croatia

**Keywords:** chronic pain, neuromodulation, neuropathic pain, post-traumatic pain, spinal cord injury, spinal cord stimulation

## Abstract

**Introduction:**

Spinal cord stimulation (SCS) is an established treatment for refractory neuropathic pain conditions, yet evidence for its effectiveness in spinal cord injury (SCI)-related neuropathic pain, particularly after decades of persistent symptoms, remains limited. It remains unclear whether unusually prolonged pain chronicity and established neurological deficits affect the potential for a meaningful response to neuromodulation.

**Case presentation:**

We report a 60-year-old man with chronic incomplete SCI, bilateral spastic paraparesis markedly more pronounced on the left, and severe chronic neuropathic pain following a gunshot-related spinal injury sustained in 1991. Retained metallic fragments were located at T12–L1; however, a formal neurological level of injury and AIS grade could not be reliably reconstructed because a complete contemporaneous ISNCSCI examination was unavailable. The longstanding bilateral lower-extremity pain phenotype was clinically consistent with below-level SCI neuropathic pain, although formal classification was limited by the absence of an ISNCSCI-defined neurological level. After more than three decades of persistent pain refractory to multimodal conservative treatment, permanent SCS was performed with two epidural leads positioned at T8–T9. Stimulation was predominantly DTM-based, supplemented by individualized tonic programming and later activation of closed-loop stimulation. Pain intensity decreased from 8/10 at baseline to 3/10 at 12 months and 2/10 at 24 months. This sustained response was accompanied by improvements in self-reported disability, neuropathic pain features, self-rated health, and psychological well-being, together with sustained discontinuation of pain-directed pharmacological therapy. No neurological deterioration or device-related complications occurred during the 24-month follow-up.

**Conclusion:**

This case demonstrates that a clinically meaningful and sustained multidimensional response to SCS can occur despite more than three decades of SCI-related neuropathic pain and established neurological impairment. Given the limited and heterogeneous evidence for SCS in this population, interpret this finding as an individual clinical observation supporting further investigation of neuromodulation in patients with longstanding SCI-related neuropathic pain, rather than as generalizable evidence for patient selection.

## Introduction

Neuropathic pain is one of the most prevalent and disabling long-term consequences of spinal cord injury (SCI), affecting approximately half of individuals after SCI and substantially impairing functional capacity, rehabilitation, psychological well-being, and quality of life (1, 2). Following traumatic neural injury, persistent neuropathic pain may coexist with permanent sensory and motor deficits and spasticity, while long-standing alterations in nociceptive processing may contribute to treatment resistance. Despite advances in pharmacological and non-pharmacological management, neuropathic pain following SCI remains challenging to treat and frequently requires an individualized, multidisciplinary approach (3, 4).

Spinal cord stimulation (SCS) is an established neuromodulatory treatment for selected patients with refractory neuropathic pain (5, 6). Its analgesic effects extend beyond the classical gate-control model and involve modulation of spinal nociceptive processing, inhibitory neurotransmission, descending pain pathways, and supraspinal networks involved in pain perception (5, 7, 8). Clinical studies and systematic reviews support meaningful and potentially sustained benefit from SCS across chronic neuropathic pain conditions, including complex regional pain syndrome, although response varies among patients and may change during long-term follow-up (9, 10).

In contrast, evidence specifically addressing SCS for neuropathic pain following SCI remains sparse. A recent systematic review demonstrated that the available literature comprises a small and heterogeneous evidence base, while successful SCS has been described in individual patients following traumatic and gunshot-related SCI (11, 12). The therapeutic potential of SCS after several decades of persistent post-traumatic neuropathic pain is even less well characterized.

We report sustained multidimensional improvement over 24 months following thoracic SCS in a patient with refractory neuropathic pain and spastic paraparesis more than three decades after gunshot-related SCI, highlighting that clinically meaningful neuromodulatory responsiveness may persist despite unusually prolonged pain chronicity.

## Case presentation

### Clinical history and preoperative assessment

A 60-year-old man was referred to our department in 2024 for evaluation of severe, treatment-refractory neuropathic pain following a remote traumatic spinal injury. In 1991, at the age of 28 years, he sustained a wartime gunshot-related spinal cord injury, with retained metallic fragments at the T12–L1 level, resulting in permanent neurological deficits, including bilateral spastic paraparesis, more pronounced on the left, left lower-limb muscle atrophy, and persistent sensory disturbances. For more than three decades, the patient experienced chronic pain involving the lower back and both lower extremities, accompanied by hypoesthesia and dysesthesia. Given the clinical characteristics and predominant bilateral lower-extremity distribution, the pain phenotype was clinically consistent with below-level SCI neuropathic pain, although formal classification was limited by the absence of a contemporaneously documented ISNCSCI-defined neurological level of injury. During the year preceding referral, pain intensity progressively increased and was accompanied by further functional deterioration. Prior to consideration of SCS, the patient had undergone prolonged physical therapy and rehabilitation and had been treated through a specialized pain clinic, where conservative pain-management strategies had been extensively pursued without adequate long-term pain control. Complete historical details of all previous pain-clinic interventions were not available for reliable retrospective reconstruction. At the time of referral for SCS, pharmacological treatment included pregabalin 150 mg three times daily and duloxetine 60 mg/day, with additional clonazepam and etoricoxib as needed. The patient had no history of diabetes mellitus, and electrodiagnostic evaluation demonstrated no evidence of peripheral neuropathy. Lumbar MSCT demonstrated no disc herniation or spinal canal stenosis requiring neurosurgical treatment, with no surgically remediable lumbar compressive lesion identified that could account for the clinical presentation. The clinical and imaging evaluation therefore did not identify a structural lumbar cause requiring surgical treatment or an alternative peripheral neuropathic process that better explained the longstanding bilateral symptoms. Musculoskeletal contributors to the overall pain burden could not be completely excluded; however, they were not considered sufficient to explain the predominant chronic neuropathic pain phenotype. Magnetic resonance imaging was contraindicated because of retained metallic fragments from the previous gunshot injury. In the context of the remote gunshot-related SCI, persistent neurological deficits and sensory disturbances, bilateral lower-extremity neuropathic pain phenotype, absence of electrodiagnostic evidence of peripheral neuropathy, absence of a surgically remediable lumbar structural lesion, and persistent symptoms despite prolonged multidisciplinary conservative pain management, the predominant chronic pain syndrome was considered attributable to the previous SCI and refractory to conservative treatment.

Neurological examination confirmed bilateral spastic paraparesis, markedly more pronounced on the left, with severe motor impairment and pronounced muscle atrophy of the left lower extremity. Lower-extremity sensory disturbances, including hypoesthesia and dysesthesia, were documented, while sacral sensory function, including anal sensation, was preserved. Despite the severe left-sided motor deficit, the patient remained independently ambulatory without walking aids, although gait was impaired by dragging of the left lower extremity; he also used a left ankle orthosis. Sphincter function was intact. Collectively, these findings were consistent with a chronic incomplete SCI. A complete contemporaneous ISNCSCI examination had not been performed; therefore, a formal neurological level of injury, segmental motor and sensory scores, and an AIS grade could not be reliably reconstructed retrospectively. Although spasticity was clinically evident, standardized quantitative grading such as the Modified Ashworth Scale had not been performed. Baseline assessment demonstrated severe pain and substantial impairment across multiple clinical domains. Pain intensity was 8/10 on the Visual Analog Scale (VAS), the Oswestry Disability Index (ODI) was 60%, and the DN4 score was 8. Self-rated health was impaired, with an EQ-VAS score of 40/100. Hospital Anxiety and Depression Scale (HADS) scores were 10 for both anxiety and depression (Figure 1). Clinical outcomes were assessed using a standardized multidimensional assessment battery at three predefined time points: before SCS implantation (baseline) and at 12 and 24 months after implantation. This multidimensional battery represents the standard clinical assessment routinely used for patients undergoing SCS at our center. At each assessment, a member of the treating clinical team provided the same set of patient-completed questionnaires, comprising the Visual Analog Scale (VAS), Oswestry Disability Index (ODI), Douleur Neuropathique 4 (DN4), the self-rated health component accompanying the EQ-5D-3L assessment, and the Hospital Anxiety and Depression Scale (HADS). The patient completed the questionnaires under clinical supervision, using the same standardized procedure at all three time points. We collected the standardized outcome measures contemporaneously at these predefined assessments and did not reconstruct them retrospectively from narrative clinical records. The VAS reflected the patient's average pain intensity over the preceding several days within the respective assessment period, rather than the worst pain experienced. Figure 1 summarizes the overall clinical course, treatment milestones, and follow-up trajectory.

**Figure 1:**
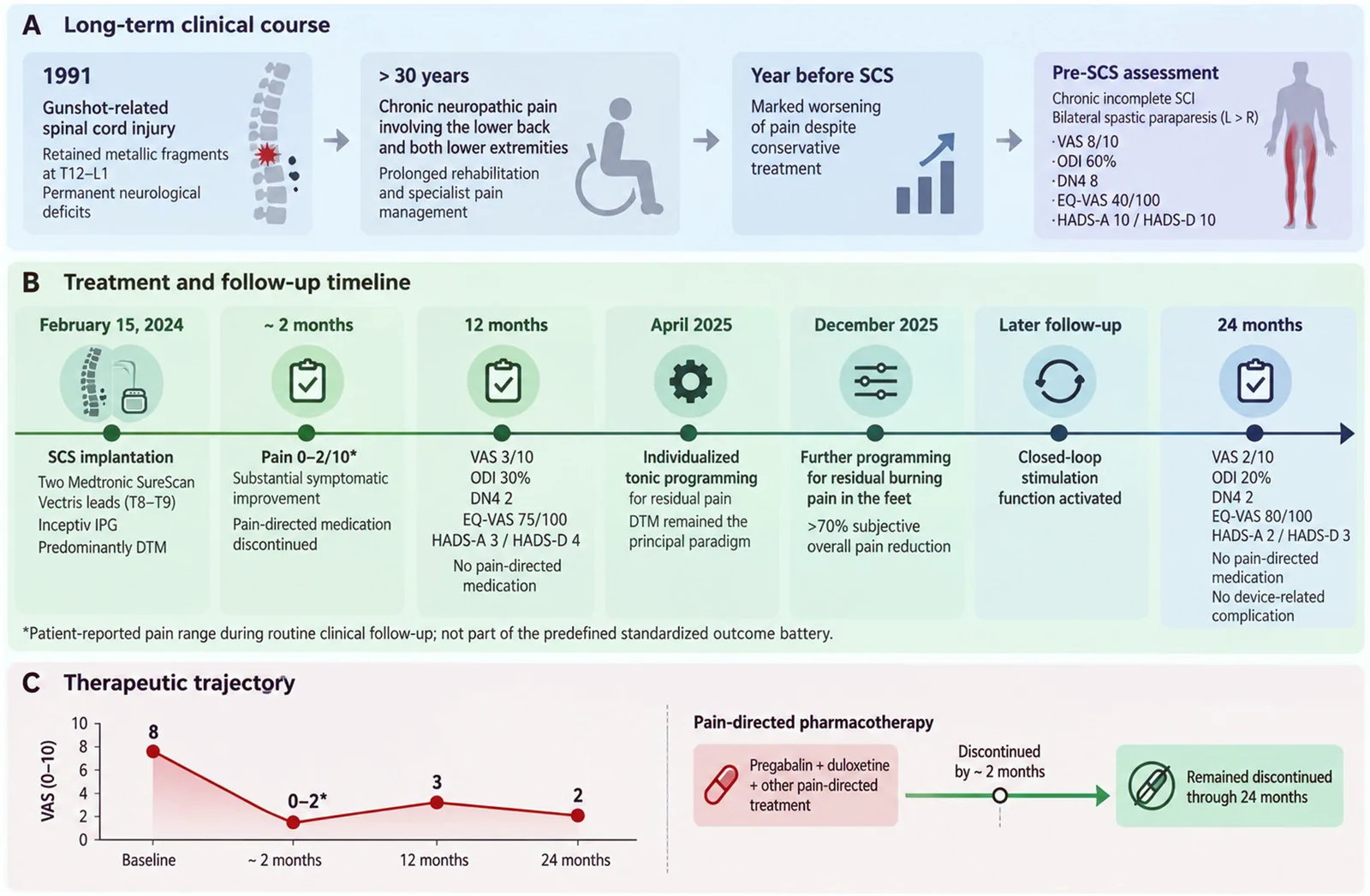
Clinical timeline and therapeutic trajectory. **(A)** Long-term clinical course from the original gunshot-related spinal cord injury in 1991 through more than three decades of chronic neuropathic pain and subsequent worsening before SCS implantation. **(B)** Timeline of SCS implantation, major programming milestones, and clinical follow-up through 24 months. Standardized multidimensional assessments were performed at baseline and at 12 and 24 months, whereas the pain range of 0–2/10 at approximately two months represents a patient-reported range documented during routine clinical follow-up and was not part of the predefined standardized outcome battery. **(C)** Temporal trajectory of pain intensity and pain-directed pharmacological treatment, demonstrating discontinuation of pregabalin, duloxetine, and other pain-directed pharmacological therapy by approximately two months after SCS implantation, with no reintroduction through 24 months. DTM, differential target multiplexed; EQ-VAS, EuroQol Visual Analogue Scale; HADS-A, Hospital Anxiety and Depression Scale–Anxiety; HADS-D, Hospital Anxiety and Depression Scale–Depression; IPG, implantable pulse generator; ODI, Oswestry Disability Index; SCS, spinal cord stimulation; SCI, spinal cord injury; VAS, Visual Analog Scale.

### Spinal cord stimulation

Following multidisciplinary assessment, and considering the predominant neuropathic pain phenotype, refractoriness to previous treatment, and absence of a surgically correctable structural lesion, the patient was selected for SCS. No separate temporary SCS trial was performed, and the patient underwent a single-stage permanent implantation. On February 15, 2024, SCS implantation was performed under local anesthesia with the patient in the prone position, allowing intraoperative stimulation testing and direct patient feedback regarding coverage of the painful areas. Under fluoroscopic guidance, two Medtronic SureScan Vectris epidural leads were advanced and positioned at the T8–T9 level, with one lead positioned more cranially and the other more caudally. Intraoperative stimulation confirmed satisfactory coverage of the painful territories, after which the leads were anchored and connected to a Medtronic Inceptiv implantable pulse generator positioned subcutaneously in the left gluteal region. The procedure was completed without perioperative complications. The postoperative course was uneventful, with no new neurological deficit, and the patient was mobilized and discharged home in good general condition. Initial programming was predominantly based on DTM stimulation, which remained the patient's principal stimulation paradigm during follow-up. A tonic stimulation program was also available, with a documented contact configuration of 12+/13−/14+, an amplitude of 3.1 mA, pulse width of 300 µs, and frequency of 50 Hz.

### Clinical outcome and 24-month follow-up

Clinical benefit was evident early after implantation. At approximately two months, during routine clinical follow-up, the patient reported substantial symptomatic improvement, with pain intensity ranging from 0 to 2/10 and markedly improved quality of life. This value represented the patient-reported range reflecting fluctuations in pain intensity during that period, documented during routine clinical assessment, and was not part of the predefined standardized outcome battery. By this time, pregabalin, duloxetine, and the remaining pain-directed pharmacological treatment had been discontinued, and the patient no longer required medication for the treated chronic neuropathic pain.

At 12 months, standardized assessment demonstrated marked improvement across all evaluated domains. Pain intensity decreased from VAS 8/10 to 3/10, ODI improved from 60% to 30%, and DN4 decreased from 8 to 2. Self-rated health improved substantially, with the EQ-VAS increasing from 40/100 at baseline to 75/100 at 12 months. Psychological well-being improved in parallel, with HADS anxiety decreasing from 10 to 3 and HADS depression from 10 to 4 (Figure 2).

**Figure 2:**
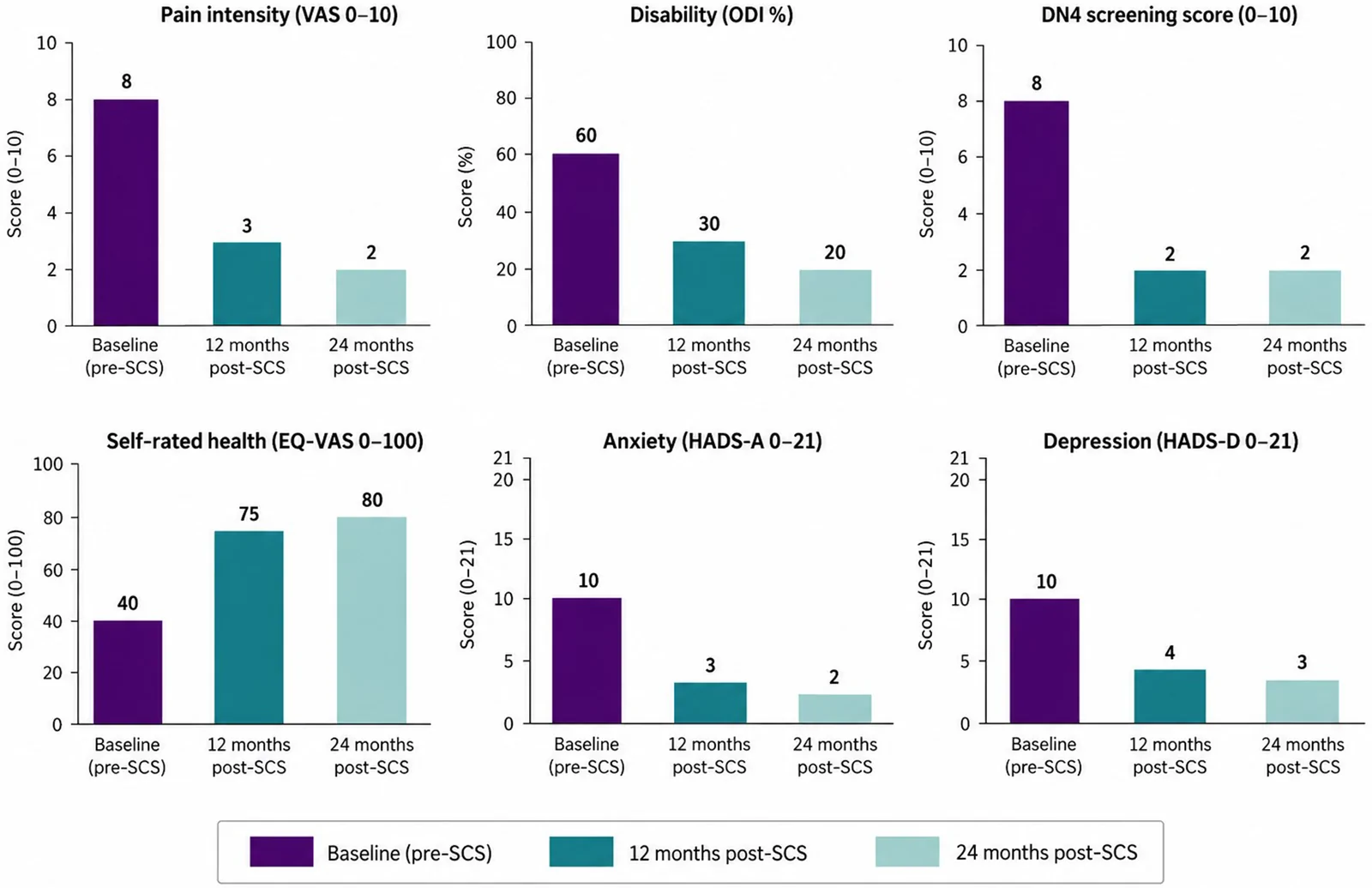
Longitudinal multidimensional clinical outcomes following spinal cord stimulation. Pain intensity (VAS), self-reported disability (ODI), DN4 screening score, self-rated health (EQ-VAS), and psychological status (HADS anxiety and depression) are shown at baseline and at 12 and 24 months following SCS implantation. Improvement was observed across the assessed domains at 12 months and was sustained or further improved at 24 months. The ODI reflects self-reported disability and should not be interpreted as a measure of neurological or motor recovery. DN4 values are presented descriptively as screening scores reflecting neuropathic pain features rather than as a validated quantitative measure of treatment response. VAS, Visual Analog Scale; ODI, Oswestry Disability Index; DN4, Douleur Neuropathique 4; EQ-VAS, EuroQol Visual Analog Scale; HADS, Hospital Anxiety and Depression Scale; SCS, spinal cord stimulation.

At the 12-month assessment, the discontinuation of pain-directed pharmacological therapy was maintained, with no reintroduction of pregabalin, duloxetine, or other medication for the treated chronic neuropathic pain. The clinical response remained sustained during subsequent follow-up. Serial clinical assessments documented a patient-reported overall pain reduction exceeding 60% relative to his preoperative condition. This percentage represented the patient's subjective estimate of overall pain improvement and was not mathematically derived from the standardized VAS assessments. This improvement was accompanied by maintained physical activity and improved quality of life. Residual burning pain, predominantly in the feet and lower legs, occurred intermittently, particularly in the evening, and was addressed through individualized adjustments to stimulation programs. Programming was individualized according to residual pain distribution and stimulation coverage, with additional tonic programs introduced during follow-up and used less frequently than DTM stimulation. In April 2025, a tonic program using contacts 13+/14− was documented at 2.2 mA, 850 µs pulse width, and 70 Hz, providing stimulation coverage of the back, buttock, entire left lower extremity, and right foot; a DTM program remained available. By December 2025, the patient subjectively estimated an overall pain reduction exceeding 70% relative to his preoperative condition, despite residual evening burning pain. At this stage, a program targeting residual burning pain in the feet used contacts 12+/13−/14−/15+, with an amplitude of 4.7 mA, a pulse width of 280 µs, and a frequency of 50 Hz. During a later follow-up phase, the Inceptiv system's closed-loop stimulation function was also activated; however, detailed ECAP-based programming parameters from that session were not available for reliable retrospective extraction. No major changes in the patient's rehabilitation regimen during follow-up could account for the observed clinical improvement, and no additional pain-directed intervention was introduced.

At 24 months, the multidimensional therapeutic response was maintained or further improved. VAS decreased to 2/10 and ODI to 20%, while DN4 remained stable at 2. Self-rated health remained improved, with the EQ-VAS increasing further to 80/100. HADS anxiety and depression scores decreased further to 2 and 3, respectively (Figure 2). At this assessment, the discontinuation of pain-directed pharmacological therapy remained sustained, with no reintroduction of pregabalin, duloxetine, or other medication for the treated chronic neuropathic pain. Occasional analgesic intake was limited to unrelated symptoms, such as headache or nonspecific general pain, which the patient explicitly distinguished from his preoperative pain condition. No neurological deterioration, infection, lead-related adverse event, or other device-related complication was observed during the 24-month follow-up.

### Patient perspective

The patient described the years preceding SCS as increasingly burdensome, with longstanding neuropathic pain substantially affecting his daily functioning and quality of life despite prolonged conservative treatment. Following SCS implantation, he reported substantial and sustained pain relief, improved quality of life, and an increased ability to remain physically active. From his perspective, a key benefit was discontinuing pain-directed pharmacological treatment. Although intermittent residual burning pain in the feet and lower legs persisted, particularly in the evening, the patient considered the overall treatment outcome highly satisfactory and reported that the improvement had remained meaningful throughout the 24-month follow-up.

## Discussion

SCS produced a marked and sustained multidimensional clinical response in our patient despite more than three decades of refractory neuropathic pain following traumatic SCI. Pain intensity decreased from a VAS score of 8/10 at baseline to 3/10 at 12 months and 2/10 at 24 months, while disability, neuropathic pain characteristics, health-related quality of life, and psychological well-being improved in parallel. Importantly, these benefits were maintained or further enhanced during the second year of follow-up and were accompanied by a sustained discontinuation of pain-directed pharmacological therapy. Although SCS is an established treatment for selected refractory neuropathic pain conditions, including CRPS, long-term response is heterogeneous and may diminish in some patients over time (9, 10, 13). The sustained response observed in the present case is therefore noteworthy in the context of the unusually prolonged duration of symptoms and coexisting permanent neurological impairment. Evidence supporting SCS specifically for neuropathic pain following SCI remains limited and considerably less robust than that available for more established SCS indications. The recent systematic review by Gallacher et al. identified only 10 eligible studies comprising 43 patients, and the available literature consists predominantly of individual case reports and small case series, with substantial heterogeneity in injury characteristics, stimulation paradigms, lead positioning, and outcome reporting (12). Overall, the certainty of evidence remains low, and the predominantly favorable outcomes reported in this small literature should be interpreted cautiously, particularly given the potential for selection and publication bias. Individual reports nevertheless demonstrate the feasibility of SCS in selected patients with traumatic SCI. Of particular relevance, Rosales et al. reported successful SCS for refractory neuropathic pain following gunshot-related incomplete SCI, providing a traumatic injury context comparable to that of our patient (11). Within this limited and heterogeneous literature, the more than three-decade interval between injury and SCS implantation in the present case represents an unusually prolonged period of pain chronification. We therefore do not interpret this case as establishing the longest reported interval, but rather as illustrating that clinically meaningful neuromodulatory responsiveness may persist despite very longstanding SCI-related neuropathic pain.

Pain duration is particularly relevant to this observation. Chronic neuropathic pain is accompanied by persistent changes in spinal and supraspinal nociceptive processing, raising the possibility that prolonged pain chronification may influence responsiveness to neuromodulation. In a systematic review and meta-regression of SCS outcomes, longer mean pain duration was associated with reduced pain relief in univariable analysis, although no patient- or technology-related factor remained independently predictive in the multivariable model (14). Our case should therefore not be interpreted as evidence that chronicity is irrelevant to patient selection; rather, it illustrates that prolonged pain duration does not invariably eliminate the capacity for a clinically meaningful and durable response. In this individual patient, a prominent neuropathic pain phenotype and the absence of a surgically correctable structural lesion supported consideration of SCS despite the unusually long duration of symptoms. The biological plausibility of such persistent responsiveness is consistent with the increasingly complex understanding of SCS mechanisms. The classical gate-control model, based predominantly on activation of large-diameter dorsal column fibers and inhibition of nociceptive transmission within the dorsal horn, represents only one component of SCS-mediated analgesia (5). Contemporary evidence implicates modulation of excitatory and inhibitory neurotransmission, dorsal horn neuronal activity, glial and neuroimmune signaling, and ascending nociceptive pathways (7). SCS also engages supraspinal networks and descending inhibitory systems involved in both sensory-discriminative and affective dimensions of pain (8, 15). These distributed effects provide a plausible framework for the parallel improvements observed in pain intensity, psychological well-being, and perceived health status in our patient. Importantly, these clinical observations cannot establish a mechanistic effect but are compatible with modulation extending beyond simple interruption of ascending nociceptive transmission.

Patient selection remains central to successful SCS therapy. Contemporary recommendations emphasize an appropriate pain indication, failure of conservative management, realistic expectations, psychological assessment, and individualized evaluation before permanent implantation (6). Because SCS is an invasive intervention involving epidural lead placement and an implantable pulse generator, the anticipated clinical benefit must be weighed against potential perioperative and device-related risks on an individual basis. In our patient, multidisciplinary assessment considered the predominantly neuropathic pain phenotype, substantial symptom and functional burden, failure of less invasive treatment strategies, absence of a surgically remediable compressive lesion, and the potential benefits and risks of neuromodulation before proceeding with implantation. The favorable response despite fixed spastic paraparesis demonstrates that substantial pre-existing motor impairment did not preclude a clinically meaningful response in this individual patient. Importantly, the reduction in ODI observed in this case should be interpreted as improvement in pain-related disability rather than evidence of neurological or motor recovery.

Technological evolution has broadened the range of available neuromodulation strategies, including high-frequency stimulation, burst paradigms, and dorsal root ganglion stimulation (13, 16). Sustained benefit with 10 kHz SCS has also been demonstrated in other neuropathic pain populations, including painful diabetic neuropathy (17). Although these findings cannot be directly extrapolated to post-traumatic SCI, they illustrate the expanding potential for individualized neuromodulation. Notably, our patient achieved a durable response with thoracic SCS using predominantly DTM-based stimulation, supplemented by individualized tonic programming and later activation of closed-loop stimulation.

The 24-month follow-up represents an important strength of this report. Device-related complications, including infection, lead migration, hardware malfunction, and loss of efficacy, remain relevant considerations during long-term SCS treatment (18). In the present case, no perioperative or device-related complication occurred, and clinical benefit was maintained throughout two years of follow-up. The sustained reduction in pain and self-reported disability, improvement in self-rated health and psychological outcomes, and sustained discontinuation of pain-directed pharmacological therapy together indicate that the observed therapeutic benefit extended beyond pain intensity alone.

Several limitations inherent to a single-patient report must be acknowledged. Causal inference cannot be established, and the observed improvements may partly reflect nonspecific treatment effects, changes in concomitant therapy, rehabilitation, or other unmeasured factors. Furthermore, favorable outcomes from an individual case should be interpreted within the context of a limited and heterogeneous SCI literature that is potentially susceptible to publication and selection bias. Moreover, the authors did not perform a formal quantitative assessment of motor recovery; therefore, they cannot conclude that neurological function was restored. The multidimensional assessment battery used in this case reflects the standard clinical framework routinely applied to patients undergoing SCS at our center; however, not all of its components are specifically validated for individuals with SCI and permanent neurological deficits. In particular, the ODI is not an SCI-specific instrument, and some of its functional items may have been influenced by the patient's permanent spastic paraparesis independently of pain. Accordingly, the observed improvement in ODI should be interpreted as a reduction in self-reported disability rather than as evidence of neurological or motor recovery. Similarly, the DN4 was developed primarily as a screening instrument for neuropathic pain rather than as a validated quantitative measure of treatment response. Changes in the DN4 score should therefore be interpreted descriptively as changes in neuropathic pain features rather than as a quantitative measure of therapeutic efficacy. Finally, although 24 months of follow-up demonstrates durability beyond the early postoperative period, longer observation is needed to determine whether the therapeutic response remains stable over subsequent years.

Nevertheless, this case expands the limited clinical experience with SCS for post-traumatic neuropathic pain following SCI. Rather than suggesting that chronicity should be disregarded, this case demonstrates that clinically meaningful neuromodulatory responsiveness can be observed even after more than three decades of refractory pain and established neurological impairment. This observation supports further investigation of symptom duration as a potential modifier, rather than an absolute determinant, of SCS responsiveness in SCI-related neuropathic pain. The sustained multidimensional response observed over 24 months further highlights the value of assessing therapeutic outcomes beyond pain intensity alone, incorporating self-reported disability, neuropathic pain features, psychological well-being, self-rated health, and changes in pain-directed pharmacological treatment.

## Conclusion

SCS was associated with sustained multidimensional benefit in a patient with refractory post-traumatic neuropathic pain persisting for more than three decades following SCI. Clinically meaningful improvements in pain, self-reported disability, neuropathic pain features, psychological well-being, and self-rated health were maintained or further improved over 24 months, together with sustained discontinuation of pain-directed pharmacological therapy. This case demonstrates that a clinically meaningful and durable response to neuromodulation can occur despite unusually prolonged pain chronicity and established neurological impairment. Given the limited and heterogeneous evidence for SCS in SCI-related neuropathic pain, these findings should be interpreted as an individual clinical observation rather than evidence of general efficacy. Further systematic investigation is required to define which patients with longstanding SCI-related neuropathic pain are most likely to benefit from SCS.

## Data Availability

The raw data supporting the conclusions of this article will be made available by the authors, without undue reservation.
